# Impact of standardized fortified donor human milk on growth parameters and biomarkers in preterm very low birth weight infants: a single-center propensity score-matched analysis

**DOI:** 10.3389/fped.2026.1851385

**Published:** 2026-07-17

**Authors:** Hui Zan, Xiaocui Zhang, Shan Ma, Hong Zhang

**Affiliations:** Pediatrics Department, East Campus, Affiliated Hospital of Xuzhou Medical University, Xuzhou, China

**Keywords:** biomarkers, growth and development, preterm infants, standardized donor human milk, very low birth weight infants

## Abstract

**Objective:**

This study aimed to evaluate the short-term clinical application effects of standardized fortified donor human milk (SF-DHM) in preterm very low birth weight (VLBW) infants, with a focus on its impact on growth parameters and relevant biomarkers, in order to provide scientific evidence for nutritional intervention strategies in cases of insufficient maternal milk supply.

**Methods:**

A retrospective single-center study design was adopted. A total of 80 preterm VLBW infants hospitalized in the Neonatal Intensive Care Unit (NICU) of our hospital from January 2022 to March 2025 were included. Based on propensity score matching (PSM), infants were 1:1 matched according to variables such as sex, gestational age at birth, birth weight, and severity of illness, resulting in 40 infants in the control group and 40 in the observation group. The control group received conventional feeding based on maternal milk supplemented with formula as needed; the observation group received SF-DHM as the main enteral nutritional source after standardized nutritional fortification. The two groups were compared in terms of in-hospital growth parameters (weight gain velocity, head circumference growth velocity, length growth velocity, length of hospital stay, and incidence of extrauterine growth restriction at discharge), nutritional biomarkers [prealbumin (PA), albumin (ALB), total protein (TP)], inflammatory markers [interleukin-6 (IL-6), C-reactive protein (CRP), tumor necrosis factor-alpha (TNF-α)], immune function markers (CD4+, CD8+, CD4+/CD8+), feeding tolerance, and incidence of complications.

**Results:**

There were no significant differences in baseline characteristics between the two groups after matching (*P* > 0.05). Intervention outcomes showed that the observation group had superior weight gain velocity, head circumference growth velocity, length growth velocity, shorter hospital stay, and lower incidence of growth restriction at discharge compared to the control group (*P* < 0.05). After 3 months of intervention, PA, ALB, TP, CD4+, and CD4+/CD8+ levels increased in both groups, while IL-6, CRP, and TNF-α levels decreased, with greater changes observed in the observation group (*P* < 0.05). The observation group also had lower rates of feeding intolerance and complications than the control group (*P* < 0.05).

**Conclusion:**

SF-DHM offers significant benefits in promoting growth, improving nutritional status, reducing inflammation, and enhancing immune function in preterm VLBW infants with limited nutritional support. As a safe, feasible, and effective enteral nutrition strategy, SF-DHM holds broad clinical application potential in NICU settings and is recommended for prioritized use in the nutritional management of preterm infants.

## Introduction

With advances in neonatal medicine, the survival rate of very low birth weight infants (VLBW, birth weight <1,500 g) has steadily improved in recent years. However, issues such as postnatal growth failure, impaired immune function, and neurodevelopmental disorders remain prevalent (1, 2), making them urgent challenges in perinatal medicine and NICU care. Nutritional support is a cornerstone in the management of VLBW infants, providing not only the foundation for growth and development but also essential protection against disease and long-term developmental impairment (3). The clinical dilemma lies in how to deliver enteral nutrition that is both safe and efficient to a group with high nutritional demands but limited tolerance. Breast milk is internationally recognized as the optimal source of enteral nutrition for preterm infants, especially VLBW infants, due to its superior nutritional composition, abundance of bioactive components, and immune-protective properties (4). Nevertheless, due to maternal factors such as delayed lactogenesis, insufficient milk volume, or mother-infant separation, a significant proportion of NICU-hospitalized VLBW infants are unable to receive an adequate supply of maternal milk (5). In this context, donor human milk (DHM) has emerged as an important alternative to maternal milk and is now widely used (6). Although DHM shares many compositional similarities with maternal milk and retains a number of bioactive substances, it is typically collected from term mothers, whose milk tends to have lower protein and caloric density compared to that of preterm mothers. Furthermore, the pasteurization process used for safety can degrade some of the biologically active components, diminishing its nutritional efficacy (7, 8), making it insufficient to meet the rapid growth and high metabolic demands of VLBW infants.

To address the lower nutrient density of DHM, the clinical practice of adding human milk fortifiers (HMF) to DHM has been implemented to increase its protein, caloric, and mineral content, resulting in what is known as “fortified donor human milk.” Building upon this, the concept of “standardized fortified donor human milk” (SF-DHM) has been proposed (9). SF-DHM involves quantitatively assessing individual nutritional needs and supplementing DHM with fortifiers at a standardized ratio to ensure balanced and stable nutrient composition while retaining as much bioactivity as possible. SF-DHM is designed to fulfill the unique needs of VLBW infants for high energy and high protein intake, while preserving breast milk's anti-inflammatory and growth-promoting properties, theoretically achieving an optimal balance between safety and efficacy. Recent studies (10, 11) have suggested that SF-DHM is superior to unfortified DHM or partial formula regimens in terms of weight gain and gastrointestinal tolerance. However, high-quality evidence regarding the systematic clinical benefits of SF-DHM in VLBW infants remains limited. Therefore, this study employed a propensity score matching (PSM) approach to retrospectively analyze the short-term clinical outcomes of SF-DHM in preterm VLBW infants, with strict control of confounding variables. The goal was to comprehensively evaluate its clinical efficacy and safety in NICU practice, thereby providing stronger evidence for early nutritional interventions in this vulnerable population and laying the groundwork for future large-scale, multicenter randomized controlled trials.

## Materials and methods

### Study design and participants

This was a single-center, retrospective observational study using propensity score matching (PSM) to evaluate the short-term nutritional intervention effects of SF-DHM on preterm VLBW infants. The study population comprised preterm VLBW infants hospitalized in the NICU of our hospital between January 2022 and March 2025. Inclusion criteria: (1) Preterm infants with gestational age < 32 weeks and birth weight <1,500 g; (2) Admitted to the NICU within 24 h after birth and received systematic treatment; (3) Enteral feeding initiated after clinical stabilization and continued with breast milk or donor milk support for ≥4 weeks; (4) Complete clinical records and follow-up data; (5) No additional nutritional support beyond the intervention (e.g., special enteral formulas, protein supplements) during monitoring. Exclusion criteria: (1) Major congenital malformations (e.g., neural tube defects, heart disease, gastrointestinal atresia); (2) Death or withdrawal of care within 24 h of birth; (3) Inability to complete continuous enteral nutrition post-birth; (4) Presence of severe infection, sepsis, or metabolic disease that may affect nutrient absorption; (5) Incomplete clinical records or major changes in nutritional strategies during the intervention. According to PSM principles, 1:1 nearest neighbor matching was performed using variables such as sex, gestational age, birth weight, delivery mode, Apgar score (12), and use of mechanical ventilation. A total of 80 cases were successfully matched, with 40 infants in both the control and observation groups. This study was approved by the Medical Ethics Committee (Approval No. LS2025091SCI). The study was conducted in accordance with the ethical principles of the Declaration of Helsinki. Informed consent was obtained and signed by the legal guardians of all enrolled infants.

## Methods

### Control group

Infants in the control group received standard NICU feeding protocols, primarily breastfeeding, with formula supplementation when milk volume was insufficient. Specific feeding details included: (1) Source of breast milk: Priority was given to maternal milk, which was refrigerated, warmed, and fed in divided doses as needed. When maternal milk was insufficient, specialized preterm formulas (e.g., Pre NAN, Similac Neosure, Enfamil Preterm) were used, selected by the attending physician based on clinical judgment. (2) No unified fortification: Breast milk was generally not fortified unless weight gain was slow, in which case human milk fortifier (HMF) was considered based on individualized physician assessment. When formula was used, it was reconstituted per manufacturer's instructions with fixed nutritional content (protein 2.0–2.5 g/100 mL, energy 75–80 kcal/100 mL), differing in nutrient advancement and weight gain from SF-DHM. (3) Feeding volume advancement: Following NICU protocol, feeding was initiated at 10–20 mL/kg/day and increased daily by 10–20 mL/kg if tolerated until full enteral nutrition was achieved. (4) Energy supplementation strategy: No standardized fortification was applied, thus protein, minerals, and vitamin supplementation were somewhat inadequate. Supplementation (e.g., iron, vitamin D) was provided orally based on growth monitoring.

### Observation group

Infants in the observation group received SF-DHM as the primary enteral nutrition source. Specific feeding management included: (1) Source of donor milk: All donor milk was provided by the hospital's human milk bank. Donors were strictly screened and tested negative for infectious diseases (e.g., HIV, HBV, HCV, syphilis). Milk was pasteurized using the Holder method (62.5 °C for 30 min) to ensure microbial safety. (2) Nutritional fortification protocol: Donor milk was fortified with standardized preterm-specific HMF to increase protein concentration to approximately 2.8–3.2 g/100 mL and energy to 80–85 kcal/100 mL, along with supplementation of calcium, phosphorus, zinc, vitamins A and D, iron, and other micronutrients. Fortifier brand was Nestlé BEBA FM85 for low-birth-weight preterm infants. Fortification concentration was dynamically adjusted according to feeding progress. (3) Feeding advancement: Initial volume started at 10–20 mL/kg/day. If no signs of intolerance (e.g., vomiting, gastric residuals, abdominal distension), volume was increased daily by 10–20 mL/kg until full enteral nutrition of 120–150 mL/kg/day was reached. Advancement was adjusted per individual tolerance, and daily assessments were made for gastric residuals, abdominal circumference, bowel movements, and bowel sounds. (4) Parenteral nutrition support: Until sufficient enteral intake was achieved, parenteral nutrition was administered in parallel, following NICU standard protocols with amino acids, lipid emulsions, and glucose. Total energy intake was adjusted daily based on weight and intake volume to ensure energy ≥110–130 kcal/kg/day and protein ≥3.5 g/kg/day. (5) Transition to maternal milk: If maternal milk supply gradually recovered, donor milk was progressively replaced by maternal milk while continuing nutritional fortification until the infant tolerated full maternal breastfeeding.

All infants received standardized care and nutritional guidance in a closed NICU environment. A dedicated clinical nutritionist calculated daily energy and nutrient intake. Feeding strategies were individualized for intolerance cases (e.g., fortification pauses, volume adjustments). To reduce bias, data collection and lab assessments were performed by third-party technicians, ensuring blinded evaluation.

### Observation indicators

1. Growth parameters: Including weight gain velocity, head circumference growth velocity, length growth velocity, length of hospital stay, and incidence of extrauterine growth restriction at discharge. Weight gain velocity=[1,000×ln(discharge weight/birth weight)]/(discharge age—age at regaining birth weight); Head circumference growth velocity=mean weekly increase in head circumference during hospitalization; Length growth velocity=mean weekly increase in body length during hospitalization; Growth restriction at discharge was assessed using Fenton growth charts (13).
2. Nutritional biomarkers: Before and after the 3-month intervention, 3 mL of venous blood was collected and centrifuged at 3,000 r/min for 15 min. Prealbumin (PA), albumin (ALB), and total protein (TP) levels were measured using a fully automated biochemical analyzer (Hitachi 7600).
3. Inflammatory biomarkers: Before and after the 3-month intervention, 3 mL of venous blood was collected and centrifuged at 3,000 r/min for 15 min. Interleukin-6 (IL-6), C-reactive protein (CRP), and tumor necrosis factor-α (TNF-α) levels were measured using enzyme-linked immunosorbent assay (ELISA).
4. Immune function indicators: Before and after the 3-month intervention, 3 mL of venous blood was collected. After anticoagulation, flow cytometry (BD FACSCanto II) was used to detect CD4+ and CD8+ T cell counts, and the CD4+/CD8+ ratio was calculated.
5. Feeding tolerance and complications: Feeding intolerance criteria: vomiting ≥3 times/day; reduced bowel sounds, abdominal distension; gastric residual volume exceeding 50% of the previous feeding and ≥3 times/day. Complications: included necrotizing enterocolitis, bronchopulmonary dysplasia, sepsis, and hospital-acquired infections.

### Statistical analysis

All data were processed and analyzed using SPSS 26.0 statistical software. Normally distributed data were expressed as ($\bar{x}\pm s$); between-group comparisons were analyzed using independent-samples *t*-tests; within-group pre- and post-intervention comparisons used paired t-tests. Categorical variables were expressed as *n* (%), and between-group comparisons were analyzed using the *χ*^2^ test. All statistical tests were two-sided, with *P* < 0.05 considered statistically significant.

## Results

### Baseline data comparison

After propensity score matching, there were no statistically significant differences between the two groups in terms of gender, gestational age, birth weight, Apgar score, or mechanical ventilation use (*P* > 0.05), indicating good comparability between the groups. See Table 1.

**Table 1:** Comparison of baseline characteristics [$\bar{x}\pm s$, *n* (%)].

Characteristic	Control (*n* = 40)	Observation (*n* = 40)	*t/x^2^*	*P*
Gender	-	-	0.202	0.653
Male	23 (57.5)	21 (52.5)	-	-
Female	17 (42.5)	19 (47.5)	-	-
Gestational age (weeks)	29.24 ± 1.43	29.45 ± 1.62	0.614	0.540
Birth weight (g)	1,246.35 ± 95.88	1,251.56 ± 90.43	0.250	0.803
5 min Apgar score	8.42 ± 0.65	8.36 ± 0.73	0.388	0.698
Mechanical ventilation	-	-	0.464	0.495
Yes	15 (37.5)	18 (45.0)	-	-
No	25 (62.5)	22 (55.0)	-	-

### Comparison of growth and development parameters

During the intervention, the observation group showed significantly better outcomes in weight gain velocity, head circumference growth velocity, and length growth velocity. They also had a shorter hospital stay and lower incidence of growth retardation at discharge (*P* < 0.05). See Table 2.

**Table 2:** Comparison of growth and development parameters [$\bar{x}\pm s$, *n* (%)].

Parameter	Control (*n* = 40)	Observation (*n* = 40)	*t/x^2^*	*P*
Weight gain velocity (g/kg/d)	12.19 ± 2.38	15.73 ± 2.44	6.568	<0.001
Head circumference growth (cm/wk)	0.64 ± 0.11	0.76 ± 0.13	4.456	<0.001
Length growth (cm/wk)	0.92 ± 0.14	1.09 ± 0.16	5.057	<0.001
Hospital stay (d)	52.37 ± 9.64	45.69 ± 8.43	3.299	0.001
Growth retardation at discharge	16 (40.0)	7 (17.5)	4.942	0.026

### Comparison of nutritional biomarkers

**Figure 1:**
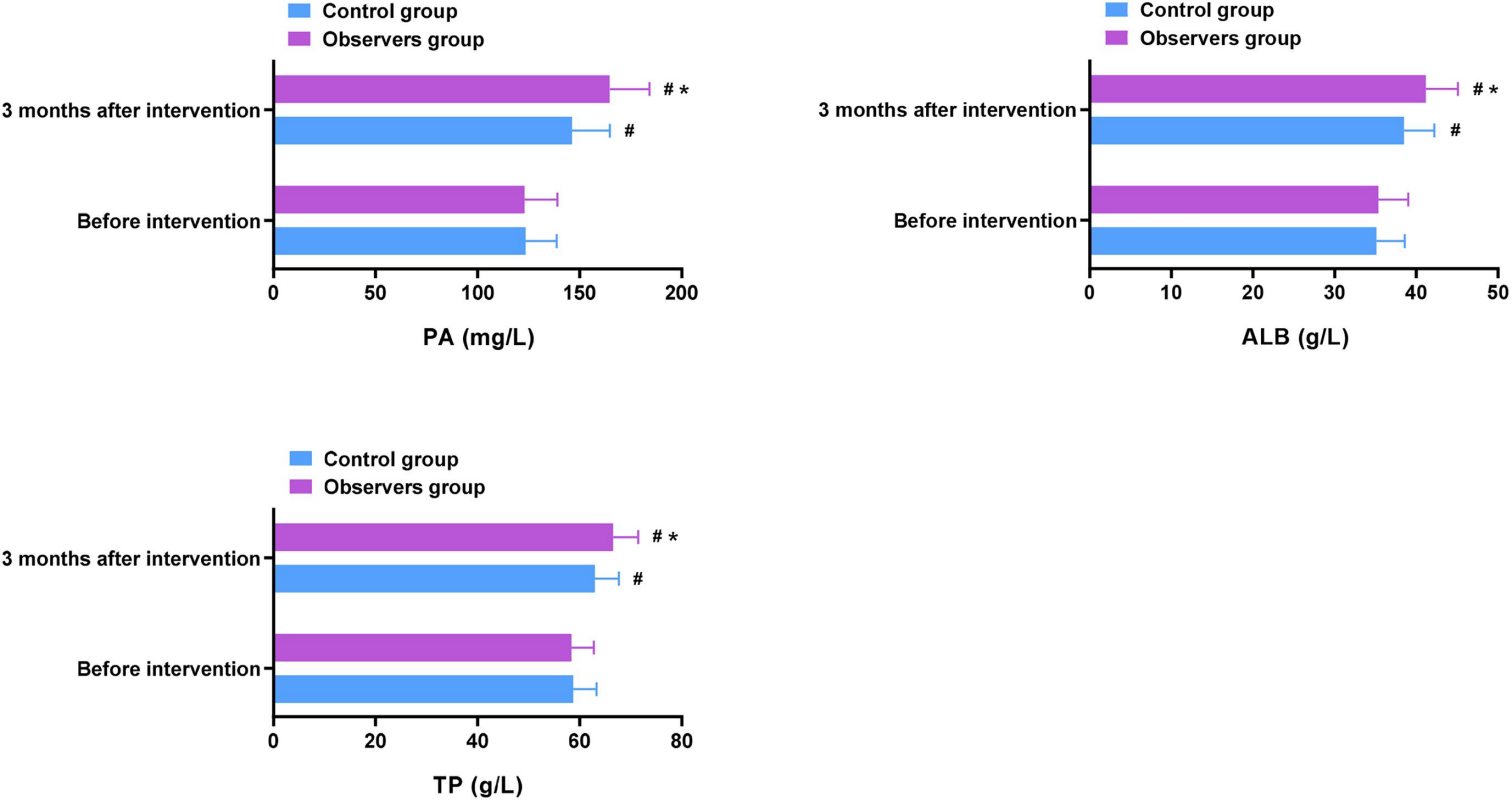
Comparison of nutritional biomarkers ($\bar{x}\pm s$). #*P* < 0.05 vs. before intervention in same group; *P < 0.05 between groups.

After 3 months of intervention, levels of PA, ALB, and TP increased in both groups compared with baseline, with a greater increase observed in the observation group (*P* < 0.05). See Figure 1.

### Comparison of inflammatory biomarkers

**Figure 2:**
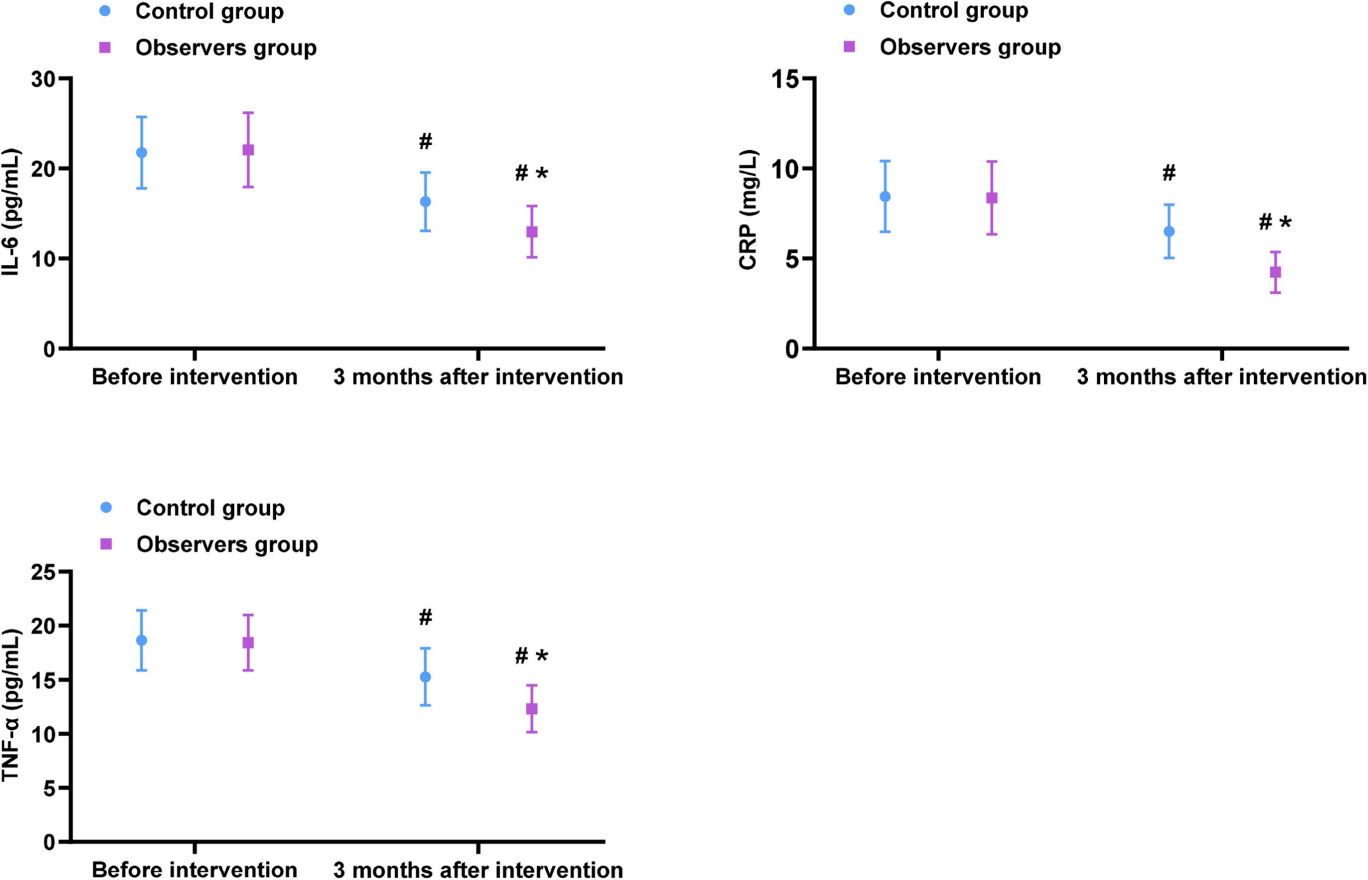
Comparison of inflammatory biomarkers ($\bar{x}\pm s$). #*P* < 0.05 vs. before intervention in same group; *P* < 0.05 *between groups.

After 3 months of intervention, levels of IL-6, CRP, and TNF-α decreased in both groups compared with baseline, with a greater reduction observed in the observation group (*P* < 0.05). See Figure 2.

### Comparison of immune function indicators

**Figure 3:**
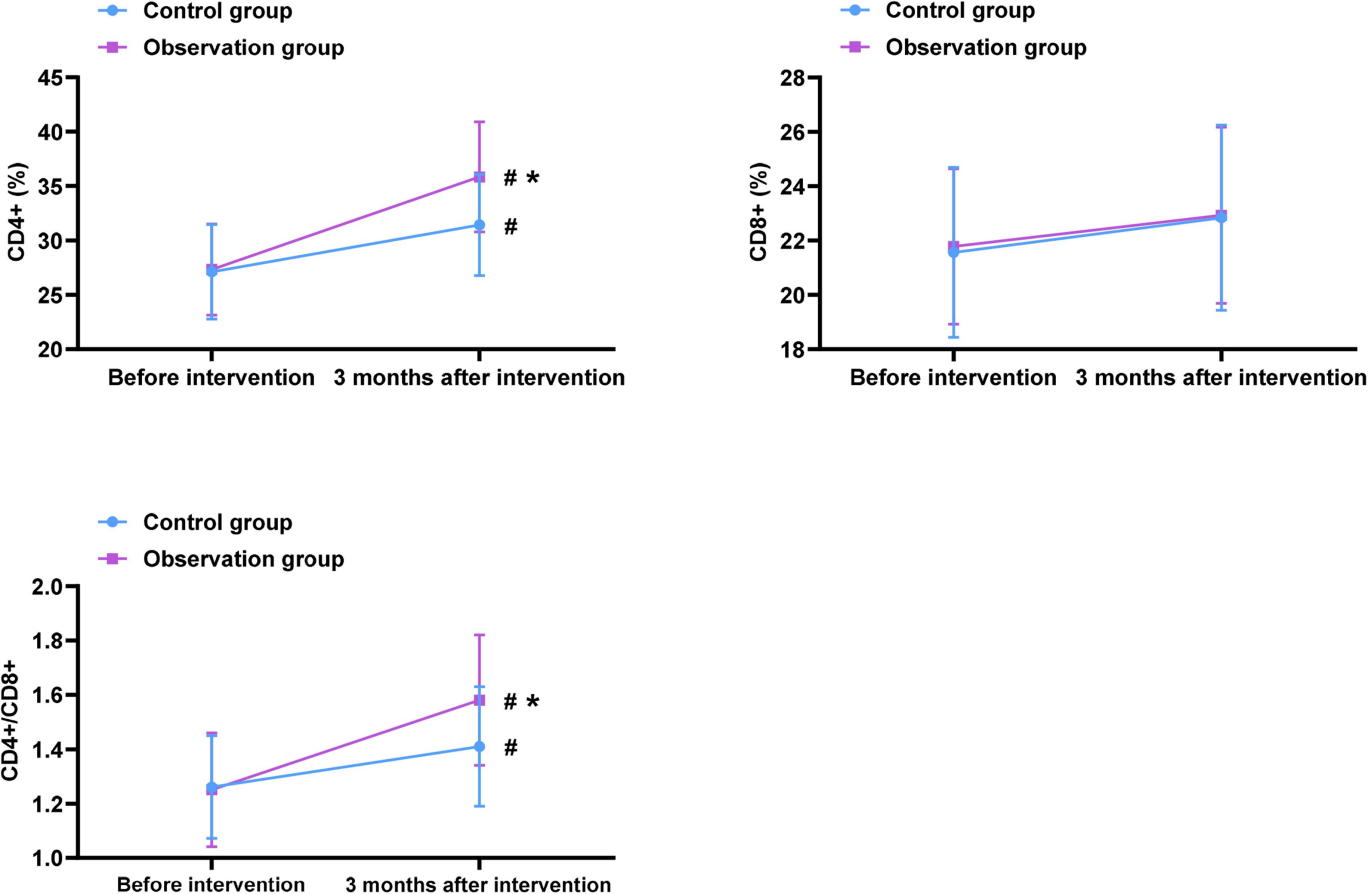
Comparison of immune function indicators ($\bar{x}\pm s$). #*P* < 0.05 vs. before intervention in same group; *P < 0.05 between groups.

After 3 months of intervention, levels of CD4+ and CD4+/CD8+ increased in both groups compared with baseline, with a greater increase observed in the observation group (*P* < 0.05). See Figure 3.

### Comparison of feeding tolerance and complications

The observation group had significantly lower rates of feeding intolerance and complications compared to the control group (*P* < 0.05). See Table 3.

**Table 3:** Comparison of feeding tolerance and complications [*n* (%)].

Outcome	Control (*n* = 40)	Observation (*n* = 40)	*x^2^*	*P*
Necrotizing enterocolitis	2 (5.0)	1 (2.5)	-	-
Bronchopulmonary dysplasia	2 (5.0)	0 (0.0)	-	-
Sepsis	1 (2.5)	0 (0.0)	-	-
Nosocomial infection	5 (12.5)	2 (5.0)	-	-
Total complication rate	10 (25.0)	3 (7.5)	4.500	0.033
Feeding intolerance rate	8 (20.0)	2 (5.0)	4.114	0.042

## Discussion

Preterm infants, especially VLBW infants, face significant metabolic and growth challenges after birth due to immature gastrointestinal morphology and function, resulting in limited autonomous intake capacity and nutrient absorption efficiency (14). Therefore, providing an enteral nutritional support strategy that balances high energy density, bioactivity, and good digestive adaptability has become a critical issue in neonatal intensive care. SF-DHM was proposed in this context. It involves analyzing the composition of donor milk and precisely adding proteins, carbohydrates, lipids, and minerals for individualized fortification, thereby meeting the physiological needs of VLBW infants while retaining the natural beneficial components of breast milk, such as lactoferrin, immunoglobulins, and epidermal growth factor, thus achieving dual functions of “nutritional support” and “immune protection” (15). This study demonstrated that SF-DHM significantly improved weight, length, and head circumference growth rates in VLBW infants, consistent with the findings of Halleux et al. (16), who reported that preterm infants fed fortified donor milk exhibited faster weight gain during the first four weeks of life and a lower incidence of complications compared to those fed conventional formula. Compared with the “individualized breast milk fortification” strategy proposed by Parat et al. (17), although the SF-DHM fortification used in this study did not fully achieve personalized customization, it still yielded favorable growth outcomes through standardized nutrient control in clinical practice, suggesting that the model of “standardization+precise addition” is clinically feasible. Moreover, the growth-promoting mechanism of SF-DHM lies not only in the quantitative sufficiency but also in the qualitative optimization. Bioactive peptides and enzymes in breast milk can promote intestinal villus proliferation and brush border enzyme expression, thereby enhancing nutrient absorption capacity (18). Additionally, the rational lactose content and milk fat structure in SF-DHM may serve as critical substrates for brain development in preterm infants, optimize gastric emptying rates, and reduce gastrointestinal discomfort related to feeding.

In terms of nutrition-related biomarkers, this study found that after 3 months of intervention, the increases in PA, ALB, and TP levels were greater in the observation group, reflecting the obvious advantages of SF-DHM in improving protein intake and synthesis. Due to its short half-life of only 2–3 days, PA is widely regarded as a sensitive indicator for monitoring short-term nutritional changes. Studies (19) suggest that increased PA levels not only reflect the timeliness of protein supply but also indicate improved intestinal function and enhanced anabolic efficiency. In contrast, ALB and TP are more indicative of hepatic synthesis capacity and chronic nutritional status; their significant increases post-intervention further demonstrate that SF-DHM can positively reshape overall nutritional status. These results are in line with findings by Karoobi et al. (20), who reported that preterm infants receiving high-protein, breast milk–based fortified feeding regimens recovered nitrogen balance earlier and exhibited markedly higher protein synthesis and tissue repair rates. It is noteworthy that, compared with infants fed traditional formula, SF-DHM-fed infants benefit from better protein composition, higher bioavailability, and natural immune-regulating factors. Although formula theoretically contains complete nutrition, its relatively inferior protein quality and absence of bioactive factors may result in lower growth efficiency and increased metabolic burden, even posing risks of protein overload and renal strain (21). On the other hand, improved protein intake has profound impacts on immune function, tissue repair, and stress response. The synthesis of immunoglobulins, acute-phase proteins, enzymes, and transport proteins requires an adequate amino acid supply (22). Nutritional improvement directly affects the synthesis rates of these key molecules, reducing susceptibility to infection, promoting tissue repair, and indirectly enhancing the preterm infant's resilience to external stressors.

Regarding inflammatory responses, this study showed that VLBW preterm infants fed with SF-DHM exhibited significantly greater reductions in serum IL-6, CRP, and TNF-α levels after 3 months of intervention, indicating a notable advantage of SF-DHM in suppressing systemic inflammation. It is well known that preterm infants are prone to a pathological state of “low-grade persistent inflammation” due to incomplete intestinal development, compromised mucosal barrier function, and delayed colonization of gut microbiota (23). This inflammatory state is closely linked to multiple preterm-related complications, including necrotizing enterocolitis and neonatal sepsis, severely affecting both short-term survival and long-term neurodevelopmental outcomes (24). The mechanisms by which SF-DHM modulates inflammatory responses can be explored on multiple levels. Firstly, its content of lactoferrin, oligosaccharides, and various milk-derived enzymes confers strong antimicrobial and immunomodulatory properties. Lactoferrin can bind to bacterial cell walls and competitively inhibit iron uptake, thereby limiting overgrowth of Gram-negative bacteria (25); it can also upregulate mucosal MUC2 expression, enhancing the integrity of the intestinal physical barrier (26). Oligosaccharides act as prebiotics to promote colonization by beneficial bacteria such as bifidobacteria and lactobacilli, aiding in gut microbiota homeostasis and reducing activation of inflammatory signaling (27). Further analysis suggests that multiple maternal-derived bioactive factors in SF-DHM can downregulate pro-inflammatory cytokine expression and release by modulating the Toll-like receptor (TLR) signaling pathway, especially the TLR4/NF-*κ*B axis. NF-*κ*B is a key transcription factor in the regulation of various inflammatory mediators, and its overactivation is closely associated with the chronic inflammatory state in preterm infants (28). SF-DHM reduces IL-6 and TNF-α synthesis by interrupting this signaling cascade, thereby reducing the inflammatory burden. This mechanism has been confirmed in *in vitro* studies by Walker et al. (29), which demonstrated that bioactive components in donor milk can modulate NF-*κ*B activity, providing a biological foundation for the anti-inflammatory effect of SF-DHM.

This study also found that the proportion of CD4+ lymphocytes and the CD4+/CD8+ ratio increased more significantly in the observation group after the intervention, reflecting the positive role of SF-DHM in the maturation of the immune system. T cell function is central to the establishment of adaptive immunity in neonates. However, due to underdeveloped thymus and poor peripheral lymphocyte mobilization, VLBW infants often present with imbalances in T cell subsets, particularly with suppressed CD4+ helper T cell function (30). The nutrient-rich composition of SF-DHM can promote T cell subset balance, induce immune tolerance, and enhance the development of gut-associated lymphoid tissue (GALT), thereby improving the overall immune defense capacity. Previous studies (31) have shown that compared to formula feeding, preterm infants fed with donor human milk demonstrate better recovery of CD4+/CD8+ ratios and lower infection rates, suggesting that donor milk has physiologically meaningful effects on immune system development.

Feeding intolerance is a common nutrition-related issue in NICU, with complex mechanisms involving delayed gastrointestinal motility, immune responses to milk components, and intestinal inflammation activation. SF-DHM, with its composition closer to natural breast milk, features naturally structured proteins of moderate molecular weight, making it less likely to trigger immune-mediated intolerance. In addition, its lower osmolality, appropriate lactose content, and smaller fat globule size contribute to faster gastric emptying, reduced risk of gastrointestinal bloating, and improved feeding adaptability. Relevant studies (32) indicate that, compared to traditional cow's milk–based formulas, breast milk and its derivatives significantly shorten the time needed for VLBW infants to achieve full enteral feeding and reduce feeding interruptions. In terms of complication prevention, SF-DHM integrates the triple advantages of nutritional support, immune regulation, and inflammation control. Its multifaceted mechanisms—including alleviating intestinal inflammation, preventing barrier disruption, enhancing systemic immune response, and improving antioxidant capacity—may underlie its effect in reducing the risk of complications such as necrotizing enterocolitis and sepsis. More importantly, SF-DHM exhibits good safety, stable sourcing, and strong controllability, offering a practical foundation for standardized implementation. It is expected to become one of the core strategies for nutritional support of preterm infants in NICU in the future.

Although this study used propensity score matching to minimize confounding bias, it remains a single-center retrospective study with certain limitations, including a relatively small sample size, short intervention duration, and the lack of inclusion of neurodevelopmental scores among outcome indicators. Moreover, there is still a lack of in-depth quantitative analysis of key nutritional and immune factors in SF-DHM, and the causal relationship between intervention and biological changes has not been fully elucidated. Future research should involve multicenter prospective cohorts, extend evaluations to 6–24 months post-discharge to assess neurodevelopment and quality of life, and incorporate techniques such as gut microbiota profiling, metabolomics, and human milk composition mapping to build an integrated pathway linking “nutritional intervention–gut–immunity–development,” thus promoting the scientific, standardized, and precision development of breast milk substitutes.

## Conclusion

The results of this study suggest that SF-DHM is a feasible enteral nutrition alternative for preterm VLBW infants when maternal breast milk is insufficient. It effectively supports improvements in key growth parameters such as body weight, head circumference, and length, significantly enhances nutritional status, reduces inflammation levels, boosts immune function, and is associated with good feeding tolerance and a lower risk of complications. Its application not only demonstrates short-term clinical benefits but also lays the foundation for long-term health outcomes. Therefore, early nutritional risk screening for preterm VLBW infants is recommended in clinical practice. When maternal milk is insufficient or fails to meet nutritional needs, standardized fortified donor milk should be prioritized, with targeted fortification interventions implemented according to individual needs to achieve safe, effective, and personalized nutritional support. Future studies should further expand into multicenter prospective designs to assess the long-term effects of SF-DHM on neurodevelopment and quality of life, and promote its standardized application in neonatal nutritional management.

## Data Availability

The original contributions presented in the study are included in the article/Supplementary Material, further inquiries can be directed to the corresponding author.
